# Bioenergetic Failure in Rat Oligodendrocyte Progenitor Cells Treated with Cerebrospinal Fluid Derived from Multiple Sclerosis Patients

**DOI:** 10.3389/fncel.2017.00209

**Published:** 2017-07-18

**Authors:** Deepali Mathur, Angela L. Riffo-Campos, Josefa Castillo, Jeffery D. Haines, Oscar G. Vidaurre, Fan Zhang, Francisco Coret-Ferrer, Patrizia Casaccia, Bonaventura Casanova, Gerardo Lopez-Rodas

**Affiliations:** ^1^Department of Functional Biology, University of Valencia Valencia, Spain; ^2^Department of Biochemistry and Molecular Biology, INCLIVA Biomedical Research Institute, University of Valencia Valencia, Spain; ^3^Laboratory of Molecular Pathology, Faculty of Medicine, University of La Frontera Temuco, Chile; ^4^Department of Neuroscience, Genetics and Genomics, Icahn School of Medicine at Mount Sinai, New York NY, United States; ^5^Hospital Clínico Universitario de Valencia Valencia, Spain; ^6^CSUR-Esclerosi Múltiple, Unitat Mixta d’Esclerosi Múltiple i Neurorregeneració del’IIS-La Fe, Hospital Universitari i Politécnic La Fe Valencia, Spain

**Keywords:** multiple sclerosis, neuromyelitis optica, myelin repair, glucose metabolism, gene expression, cerebrospinal fluid, oligodendrocyte progenitor cells

## Abstract

In relapsing-remitting multiple sclerosis (RRMS) subtype, the patient’s brain itself is capable of repairing the damage, remyelinating the axon and recovering the neurological function. Cerebrospinal fluid (CSF) is in close proximity with brain parenchyma and contains a host of proteins and other molecules, which influence the cellular physiology, that may balance damage and repair of neurons and glial cells. The purpose of this study was to determine the pathophysiological mechanisms underpinning myelin repair in distinct clinical forms of MS and neuromyelitis optica (NMO) patients by studying the effect of diseased CSF on glucose metabolism and ATP synthesis. A cellular model with primary cultures of oligodendrocyte progenitor cells (OPCs) from rat cerebrum was employed, and cells were treated with CSF from distinct clinical forms of MS, NMO patients and neurological controls. Prior to comprehending mechanisms underlying myelin repair, we determine the best stably expressed reference genes in our experimental condition to accurately normalize our target mRNA transcripts. The *GeNorm* and *NormFinder* algorithms showed that mitochondrial ribosomal protein (*Mrpl19)*, hypoxanthine guanine phosphoribosyl transferase (*Hprt*), microglobulin β2 (*B2m*), and transferrin receptor (*Tfrc*) were identified as the best reference genes in OPCs treated with MS subjects and were used for normalizing gene transcripts. The main findings on microarray gene expression profiling analysis on CSF treated OPCs cells revealed a disturbed carbohydrate metabolism and ATP synthesis in MS and NMO derived CSF treated OPCs. In addition, using STRING program, we investigate whether gene–gene interaction affected the whole network in our experimental conditions. Our findings revealed downregulated expression of genes involved in carbohydrate metabolism, and that glucose metabolism impairment and reduced ATP availability for cellular damage repair clearly differentiate more benign forms from the most aggressive forms and worst prognosis in MS patients.

## Introduction

Multiple sclerosis (MS) is a debilitating disease of the central nervous system (CNS) affecting the quality of life in mainly young people. Inflammatory demyelination, axonal degeneration and gliosis constitute major pathological hallmarks of MS. Although there are a variety of pharmaceutical drugs available to prevent relapses and slow down progression in MS, lack of proper understanding of its pathogenesis poses a great challenge for a complete cure of disease. During the relapsing-remitting MS subtype (RRMS), the patient’s brain itself is capable of repairing the damage, remyelinating the axon and recovering the neurological function. Furthermore, CSF is in close proximity with brain parenchyma and contains proteins and other factors, which may influence the cellular physiology of brain cells. Indeed, CSF is a promising biofluid in the search for biomarkers and disease associated proteins in MS, both with respect to inflammatory and neurodegenerative processes. Studies revealed that exposure of human CSF in xenogeneic models cause neurotoxicity in culture, although the molecular mechanisms remained poorly understood (Xiao et al., 1996; Alcázar et al., 2000). Our group found that ceramides present in CSF derived from MS patients impair neuronal bioenergetics in rat neuronal cultures (Vidaurre et al., 2014). Increased energy requirement following Na+/K+ ATPase redistribution in demyelinated axons (Waxman, 2006) and disturbed metabolic or trophic support due to damaged oligodendrocytes (Lee et al., 2012) is imputable to axonal injury which causes irreversible permanent neurological deficits in MS patients.

Several lines of evidence have indicated that metabolic disturbances contribute to the pathogenesis of neurodegenerative diseases including Alzheimer’s and Huntington’s disease (Mazzola and Sirover, 2001; Senatorov et al., 2003; Soucek et al., 2003; Blalock et al., 2004; Brooks et al., 2007; Lin et al., 2009) or MS (Jones et al., 1950; Smith and Lassmann, 2002; Mahad et al., 2008; Iñarrea et al., 2013; Broadwater et al., 2011; Safavizadeh et al., 2013; Mathur et al., 2014). Furthermore, a number of gene expression studies have been conducted in peripheral mononuclear white blood cells (Der et al., 1998; Wandinger et al., 2001; Bomprezzi et al., 2003; Koike et al., 2003; Hong et al., 2004), in MS brain tissues (Becker et al., 1997; Whitney et al., 1999; Chabas et al., 2001) and in CSF (Brynedal et al., 2010). In amyotrophic lateral sclerosis (ALS), a neurodegenerative disorder, mitochondrial deformities have been reported by several studies (Afifi et al., 1966; Atsumi, 1981; Sasaki and Iwata, 1996; Siklós et al., 1996). Furthermore, several investigations have revealed changes in the mitochondrial electron transport chain of ALS patients. These include decreased complex I activity and cytochrome c oxidase activity in skeletal muscle, spinal cords and motor cortex of ALS patients (Bowling et al., 1993; Fujita et al., 1996; Wiedemann et al., 1998; Borthwick et al., 1999). ALS patients who are in their benign stage showed an elevated oxidative phosphorylation capacity of skeletal muscle mitochondria whereas patients in their progressive stage showed a significantly reduced activity of muscular mitochondrial respiratory complex IV (Echaniz-Laguna et al., 2006). These studies suggest perturbation in mitochondrial-associated pathways and eventually a variation in energy generation process. Gonzalez de Aguilar et al. (2005) have shown that the rate of glucose uptake, oxygen consumption, and lactate formation is increased significantly in skeletal muscles of ALS patients indicating disturbance in glucose metabolic pathways. Moreover, the levels of glucose within the CNS are significantly decreased in ALS patients (Dalakas et al., 1987). Apart from identifying defects in glucose metabolism in ALS, Dodge et al. (2013) found abnormalities in glycogen metabolism in spinal cords of ALS patients. All of these case studies reveal significant variations in carbohydrate metabolism in ALS, which may be related to the underlying pathology of ALS.

In support of human studies, animal mouse models of ALS have also displayed metabolic dysfunctions. In particular, mutant SOD1 mice model contains a modified structure of mitochondria, as observed in motor neurons (Dal Canto and Gurney, 1995; Wong et al., 1995; Sasaki and Iwata, 1996; Kong and Xu, 1998). Furthermore, the activity of complex I of electron transport chain is significantly reduced in SOD1G93A mice suggesting mitochondrial abnormality (Jung et al., 2002; Mattiazzi et al., 2002). Other investigations have also revealed impaired electron transport chain and defects in ATP synthesis in spinal cords of mutant SOD1 mice (Mattiazzi et al., 2002). It was found that glucose levels and ATP generation declined to a great extent within CNS tissues in SOD1G93A mice (Browne et al., 2006). Other neurodegenerative disorders, such as Alzheimer’s disease, have also shown aberrations in mitochondrial DNA and in associated enzymes (Zhu et al., 2006). In this study, mitochondrial DNA and cytochrome oxidase-1 levels are elevated in hippocampal neurons, compared to control brains, even though the number of mitochondria per neuron is declined. Altogether, evidence from these studies indicates that glucose metabolism, oxidative balance, and ATP production are extensively impaired in ALS and other neurodegenerative diseases.

Findings from our previous work demonstrated that *Gapdh*, a commonly used reference gene, showed downregulated expression when cerebellar granule neurons were treated with CSF obtained from distinct clinical types of MS and NMO patients (Mathur et al., 2015). *Gapdh* is a key glycolytic enzyme involved mainly in the production of ATP. Standard reference genes such as *Gapdh* and b*-actin* have been demonstrated to show variable expression in different experimental conditions (Zhong and Simons, 1999; Torres et al., 2003; Toegel et al., 2007; Gubern et al., 2009; Nelissen et al., 2010). We therefore, investigated the expression stability of six reference genes in OPCs treated with CSF from MS and NMO patients, using *geNorm* and *NormFinde*r algorithms. Same reference genes that we studied previously in neuronal analysis (Mathur et al., 2015) were selected. *geNorm* program defines the gene stability as the average pairwise variation of a particular gene with all other reference genes and ranks the genes according to their average expression stability (*M*). The gene with minimum *M*-value is considered to be highly stable whereas the gene with highest *M*-value is least stable. The *geNorm* algorithm is based on the assumption that the reference genes selected for analysis are not co-regulated. Keeping this in mind, we selected genes that were regulated differently in order to elude unbiased results. Another program, *NormFinder*, ranks the candidate reference genes based on the combined estimates of both intra- and intergroup variations. In general, more than one reference genes are recommended to use for the correct normalization of gene expression data (Zhong and Simons, 1999; Hamalainen et al., 2001; Tricarico et al., 2002; Vandesompele et al., 2002; Ohl et al., 2005).

The goal of the present study was to investigate the effect of CSF on OPCs, which could contain factors that damage OPCs during attempts at brain repair. Additionally, we wanted to assess the expression stability of various commonly used reference genes when rat OPCs were treated with CSF from MS and NMO patients. This step would enable us to accurately normalize target mRNA transcripts in gene expression experiments.

## Materials and Methods

### Study Approval

All procedures were approved by the Committee of Animal Care of Prince Felipe Research Center (CIPF, Valencia,) in accordance with the regulations of the European Union and Spanish legislations, within the expedient PS09/00976 of the Institute of Health Carlos III. Written informed consent was obtained from all the patients and authorized by the Ethical Committee of the Hospital Universitario y Politecnico La Fe and Hospital Clínico Universitario de Valencia for this research (Mathur et al., 2015).

### Patient Cohort

A total of 59 patients were recruited and CSF samples were obtained from the Department of Neurology, Hospital Universitario y Politécnico La Fe and Hospital Clínico Universitario de Valencia. Out of 59 patients, 21 had inflammatory MS (11 G+/M+ and 10 G+/M-), 8 had medullary subtype (Med), 11 had primary progressive MS (PPMS), 9 had NMO, and 10 were non-inflammatory neurological controls (NIND patients) (**Table 1**). In CSF, apart from factors related to MS or NMO, there are factors from other diseases that produce their action. This must be considered as “*background noise*” as average population. Mixing of total CSF samples in all clinical forms may potentiate the factors related to MS. Therefore, samples from patients suffering from the same form of MS (e.g., G+/M-, G+/M+, medullary, PPMS or controls) were pooled together, then several OPCs cultures were treated with the CSF mix, and the RNA were extracted from the culture (see below).

**Table 1:** Clinical characteristics of the patients.

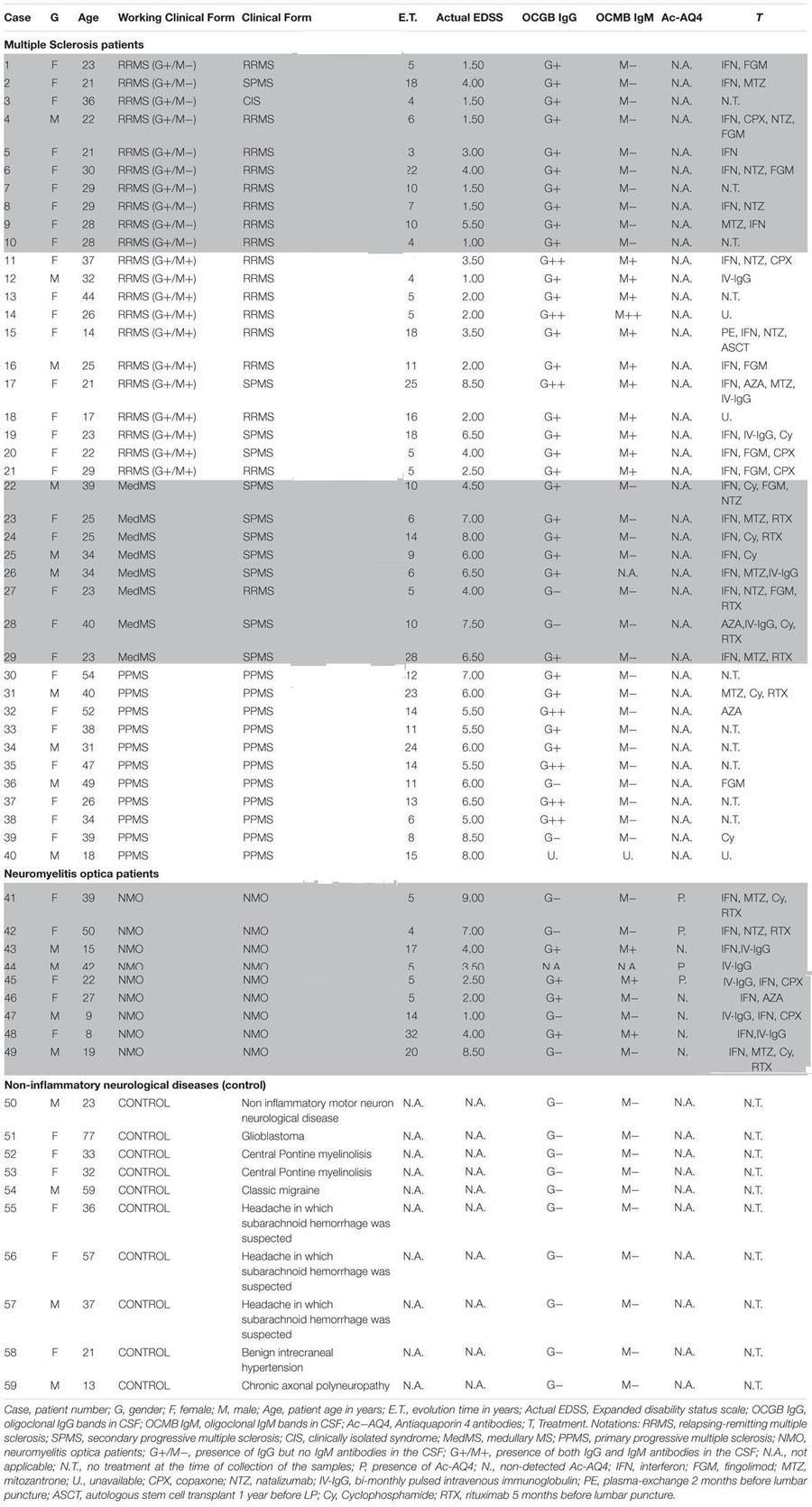

Multiple sclerosis patients were defined and grouped in different clinical courses, according to the current criteria (Lublin and Reingold, 1996) and diagnosed according to McDonald criteria. They all met the following characteristics: oligoclonal IgG bands (OCGB) present, not in a phase of relapse, and have spent more than a month after the last dose of steroids. Wingerchuk et al. (2006) criteria were used to diagnose patients with NMO disease. Patients suffered relapses of optic neuritis and myelitis, and two of the three criteria, normal MRI or that did not accomplish the Patty criteria for MRI diagnosis of MS.

### Patient Characteristics

Inflammatory MS (RRMS and SPMS forms): MS is categorized into: (1) Relapsing remitting MS (RRMS) that later develops into secondary progressive stage (SPMS); and (2) primary progressive MS (PPMS). Over 95% of patients with MS show oligoclonal bands (OCBs) of IgG in CSF (G+) (Kostulas et al., 1987) and 40% show IgM OCBs in CSF (M+) related to a more aggressive course of disease (Sharief and Thompson, 1991). In this study we also classified and named inflammatory MS into “*G*+*/M*-” and “*G*+*/M*+” subtypes (see below) on the basis of aggressivity and prognosis that is more complete than just RRMS or PPMS. In addition, we have studied separately a set of patients with MS but with a predominant affectation of the spinal cord, because these patients have some peculiarities, and we wanted to explore if they have some differences in light of our experiments. The most aggressive cases termed as “*medullary*” have more spinal injuries.

Relapsing-remitting multiple sclerosis (RRMS) IgG+/IgM- clinical form of MS: Patients named as “*G*+*/M-subtype*” had IgG antibodies (+) but no IgM (-) oligoclonal antibodies detected in the CSF of brain. RRMS IgG+/IgM+ clinical form of MS: Patients named as “*G*+*/M*+ *subtype*” had both IgG antibodies (+) and IgM (+) oligoclonal antibodies detected in the CSF of brain. Medullary (Med) clinical form of MS: All these patients were positive for OCGBs and negative (most frequent) or not for oligoclonal IgM bands (OCMBs) in CSF as well as presence of diffuse hyperintensity signal in the spinal cord and with mainly relapses from this location. The patients accomplished also Swanton’s criteria for dissemination in time. Primary progressive MS (PPMS): These patients are characterized by progressive decline in neurological disability. Neuromyelitis optica (NMO) patients: Individuals that met at least two of the following three features. (1) Long extensive transverse myelitis (>3 vestibule bodies); (2) Antibodies against aquaporin-4; (3) Normal brain at the first event Controls [Non-Inflammatory Neurological Diseases (NIND)]: Individuals who were suspected to have MS but after protocolized analysis were not diagnosed with MS.

### Cerebrospinal Fluid Samples of Patients

Cerebrospinal fluid samples were obtained by lumbar puncture at the time of diagnosis. No patient had received treatment with immunosuppressive drugs, immunomodulators or corticosteroids for at least 1 month prior to the extraction of CSF. The routine clinical extraction of CSF from patients was 10 mL obtained by lumbar puncture in subarachnoid space under sterile conditions every 3–6 months. The samples were centrifuged for 10 min at 700 × *g* and aliquots were frozen and stored at -80°C in 1 ml aliquots until use. To preserve the integrity of the samples, the aliquots were used just once in an experiment without re-frozen.

### Primary Cultures of OPCs from the Neonatal Rat Brain

The OPCs were isolated from the cortex of postnatal day 1 Wistar rats (Harlan Iberica) and grown for 1 week as mixed glial cultures according to a modified McCarthy and deVellis procedure (McCarthy and de Vellis, 1980). Differential shaking, followed by magnetic beads immunoselection, was used to isolate progenitors (Haines et al., 2015). Briefly, cells were incubated with the A2B5 antibody, purified using magnetic beads (Miltenyi Biotec), and then plated at a density of 2 × 10^5^ cells/ml on poly-D-lysine coated 10 cm plates. OPC cells were allowed to proliferate for 48 h in chemically defined media (ODM) supplemented with growth factors (GFs) [20 ng/mL basic fibroblast growth factor (bFGF) and 10 ng/mL platelet-derived growth factor AA (PDGF-AA)] prior to treatments indicated below. This procedure led to a 95% pure population of A2B5+ cells that expressed *Myelin* and *GalC*.

### CSF Treatment and RNA Extraction

Treatment of OPC was conducted by culturing the cells in a 1:1 dilution of CSF and chemically defined medium supplemented with growth factors to allow a total concentration of 20 ng/ml bFGF and 10 ng/ml PDGF-AA. In these conditions OPC remain proliferative and this does not interfere with their survival or differentiation. We performed an MTT cellular toxicity assay to determine whether CSF-exposed OPCs were viable, and no difference was detected amongst the CSF groups tested (*p* > 0.05) (Haines et al., 2015).

The RNA was extracted from OPCs treated cells using the Qiagen RNeasy RNA extraction kit according to the manufacturer’s instructions. The RNA concentration was determined spectrophotometrically at 260 nm using the Nanodrop 1000 spectrophotometer (V3.7 software) and the quality of every RNA sample was measured by means of the absorbance ratio at 260/280 nm and by capillary electrophoresis using 2100 Bioanalyzer instrument (Agilent). For microarray assays (see below), we used samples with OD (*A*_260_/*A*_280_) ratio value > 1.8 and an OD (*A*_260_/*A*_230_) ratio > 1.9 and a minimum RNA Integrity Number (RIN) score of 9.8 (mainly 10) in the Total RNA Nano Series.

### Selection of Reference Genes

We selected a panel of reference genes from microarray data that we used in neuronal analysis (Mathur et al., 2015), to normalize expression of target mRNA transcripts. From the microarray data we determined the changes of gene expression in the different MS and NMO patients, and evaluated their expression stability using *geNorm* and *NormFinde*r algorithms. Candidate reference genes, included β-actin (*ActB*), hypoxanthine guanine phosphoribosyl-transferase (*Hprt*), mitochondrial ribosomal protein L19 (*Mrpl19*), transferrin receptor (*Tfrc*), microglobulin β2 (*B2m*), and glyceraldehyde-3-phosphate-dehydrogenase (*Gapdh*), were selected for evaluation. The function and references of the genes are listed in **Table 2**. Primer sequences and amplification summary are listed in **Table 3**.

**Table 2:** Panel of six candidate housekeeping genes selected for expression analysis.

Gene symbol	Gene name	mRNA accession number	Function	Reference
*ActB*	β-Actin	NM_031144	Cytoskeletal structural Protein	Stürzenbaum and Kille, 2001
*Hprt*	Hypoxanthine guanine phosphoribosyl transferase	NM_012583	Metabolic salvage of purines	Everaert et al., 2011
*Mrpl19*	Ribosomal protein L19	NM_031103	Unclear	Zhou et al., 2010
*Tfrc*	Transferrin receptor	NM_022712	Iron delivery from transferrin to cells	Gorzelniak et al., 2001
*B2m*	Microglobulin-β-2	NM_012512	Major histocompatibility complex class I	Yurube et al., 2011
*Gapdh*	Glyceraldehyde-3-phosphate-dehydrogenase	NM_017008	NAD+ dependent glyceraldehyde-3-phosphate dehydrogenase	Gorzelniak et al., 2001; Fort et al., 1985; Harrison et al., 2000; Medhurst et al., 2000

**Table 3:** Primer sequences and amplification summary.

Gene	Primer Sequence (5′→3′)	Product size
*Actb*	F: ATTGAACACGGCATTGTCAC R: ACCCTCATAGATGGGCACAG	294
*Hprt*	F: CCTCTCGAAGTGTTGGATACAG R: TCAAATCCCTGAAGTGCTCAT	105
*Rpl19*	F: ACCTGGATGCGAAGGATGAG R: CCATGAGAATCCGCTTGTTT	139
*Ldha*	F: AGGAGCAGTGGAAGGATGTG R: AGGATACATGGGACGCTGAG	214
*Tfrc*	F: GTTGTTGAGGCAGACCTTCA R: ATGACTGAGATGGCGGAAAC	112
*B2m*	F: GTCGTGCTTGCCATTCAGA R: ATTTGAGGTGGGTGGAACTG	116
*Gapdh*	F: GGAAACCCATCACCATCTTC R: GTGGTTCACACCCATCACAA	200

*F, forward primer; R, reverse primer. Annealing temperature was 60° in whole amplicons.*

### Determination of Reference Gene Expression Stability

To determine the stability of these genes, we employed publicly available software tools named *geNorm* and *NormFinder*. A statistical test was applied to look for significant differences between experimental conditions for each candidate reference gene. A one-way analysis of variance (ANOVA) was conducted to determine the significantly variable genes. A *p*-value < 0.05 was considered statistically significant.

### Gene Microarray, Data Normalization and Gene Validation

Isolated RNA from OPC treated cells were subjected to one color microarray-based gene expression analysis (Agilent Technologies). The labeled cRNA was hybridized to the Agilent SurePrint G3 Rat GE 8x60K Microarray (GEO-GPL13521, *in situ* oligonucleotide), according to the manufacturer’s protocol. We used four microarrays per MS type of patients. Briefly, the mRNA was reverse transcribed in the presence of T7-oligo-dT primer to produce cDNA. cDNA was then *in vitro* transcribed with T7 RNA polymerase in the presence of Cy3-CTP to produce labeled cRNA. The labeled cRNA was hybridized to the Agilent Sure Print G3 Rat GE 8x60K Microarray according to the manufacturer’s protocol. The arrays were washed, and scanned on an Agilent G2565CA microarray scanner at 100% PMT and 3 μm resolution. The intensity data was extracted using the *Feature Extraction Software* (Agilent). 75th percentile signal value was used to normalize Agilent one-color microarray signals for inter-array comparisons. After normalization, the data was filtered in order to exclude probesets with low expression and/or affected by differences between the laboratories.

Differentially expressed genes were identified by comparing average expression levels in MS and NMO cases and controls. mRNA expression (in terms of absolute fold change) in OPCs treated with CSF of MS and NMO individual patients was compared with gene expression in OPC exposed to CSF from neurological control patients. Fold change cut off was considered as 2. The microarrays data correspond to at least 3–4 independent assay per group of individual MS or NMO patients and controls.

### Statistical Analysis

Statistical analysis was conducted after background noise correction using *NormExp* method. Differential expression analysis was carried out on non-control probes with an empirical Bayes approach on linear models (Smyth, 2004). Results were corrected for multiple testing hypothesis using false discovery rate (FDR), and all statistical analyses were performed with the *Bioconductor project*^1^ in the *R statistical environment*^2^. To filter out low expressed features, the 30% quartile of the whole array was calculated, and probe sets falling below threshold were filtered out. After merging the probes corresponding to the same gene on the microarray, the statistical significance of difference in gene expression were assessed using a standard one-way ANOVA followed by Tukey’s HSD *post hoc* analysis (cut-off *p* < 0.01 and FDR <0.1). All data processing and analysis including PCA plot was carried out using R functions.

### Analysis of Gene–Gene Interaction Networks Using String v10 Software

We used STRING v10 software and correlated the gene interaction in different disease subtypes in OPCs (Szklarczyk et al., 2015; STRING database)^3^. We defined a parameter to “*integrate*” our data as “*Cumulative Flux Index* or *CFI*” within the network to compare our experimental MS conditions.

The values mean that the reduction of local flux due to the inhibition of an enzymatic activity in a specific gene affected synergically to the whole metabolic flux network. It means that as more genes were down regulated, the total flux was reduced as a cumulative factor that we integrated as the total CFI of the network.

## Results

### Demographic and Clinical Profiles of MS, NMO, and NIND Groups

Baseline characteristics of the study population are described in **Table 4**. Prevalence of MS was found more in women (75%) than in men. Mean age of MS patients was 30.7 ± 9.7 years whereas 25.6 ± 15 years for NMO patients. According to the classical clinical classification, that only takes care of the general characteristics of MS patients, and classified as RRMS, SPMS, and PPMS, there were significant differences observed in age at the beginning of PPMS and the other two MS forms (*p* < 0.003); Expanded Disability Status Scale (EDSS) of RRMS and the two other MS forms (*p* < 0.001); evolution time from the first to the second episode between PPMS and RRMS (*p* = 0.043) (**Table 5**). In patients experiencing a progressive course, evolution time was similar in secondary progressive cases and in cases that were progressive from onset (13.5 vs. 13.8) (**Table 5**).

**Table 4:** General characteristics of series studied.

	Controls (*n* = 10)	MS patients (*n* = 40)	NMO patients (*n* = 9)	*P*
% females (*n*)	60.0 (6)	75.0 (30)	55.6 (5)	0.40 (χ^2^)
Age (mean, *SD*)	40.3 (19.5)	30.7 (9.7)	25.6 (15.0)	0.04 (Anova test)
EDSS	NA	4.5 (2.3)	4.6 (2.8)	0.94 (*t*-test)
Evolution time	NA	11.1 (6.6)	11.8 (9.7)	0.79 (*t*-test)

**Table 5:** Characteristics of MS patients according to clinical classification.

	RRMS (*n* = 18)	SPMS (*n* = 11)	PPMS (*n* = 11)	*P*
% females (*n*)	83.3 (15)	72.7 (8)	63.6 (7)	0.48 (χ^2^)
Age (mean, *SD*)	27.3 (7.2)	27.9 (7.3)	38.9 (11.2)	0.003 (Anova test)
EDSS	2.4 (1.2)	6.2 (1.5)	6.3 (1.1)	<0.001 (Anova test)
Evolution time	8.1 (5.4)	13.5 (7.8)	13.8 (5.7)	0.043 (Anova test)

In the **Table 6** we organize the MS patients paying attention to data we obtained in which the presence of CSF-restricted IgM OCB was associated with an active inflammatory disease phenotype in PPMS patients with more active inflammatory disease (Villar et al., 2014). With this working classification, we found significant differences in the age at beginning between PPMS and the other two MS forms (RRMS and SPMS) (*p* < 0.003) (**Table 6**). People with PPMS are usually older at the time of diagnosis, with an average age of 40. Furthermore, different subtypes of MS help to predict disease severity and response to treatment hence their categorization is important. Moreover, we found significant differences in EDSS between RRMS and the two other MS forms (SPMS and PPMS) (*p* < 0.001) (**Table 6**). Although nerve injury always occurs, the pattern is specific for each individual with MS. Disease severity and disability increases from RRMS to SPMS course and in PPMS subtype, symptoms continually worsen from the time of diagnosis rather than having well-defined attacks and recovery. PPMS usually results in disability earlier than relapsing-remitting MS.

**Table 6:** Characteristics of MS patients according to new proposal and working classification.

	RRMS and SPMS			PPMS (*n* = 11)	*p*
	Inflammatory MS (*n* = 21)		Medullar MS (*n* = 8)		
	G+/M- (*n* = 10)	G+/M+ (*n* = 11)			
% females (*n*)	90 (9)	81.8 (9)	62.5 (5)	63.6 (7)	0.40 (χ^2^)
Age (mean, SD)	26.7 (4.8)	26.3 (8.7)	31.4 (7.0)	38.9 (11.2)	0.005
EDSS	2.5 (1.5)	3.4 (2.2)	6.2 (1.4)	6.3 (1.1)	<0.001
Evolution time	8.9 (6.3)	10.8 (5.9)	8.5 (3.1)	13.8 (5.7)	0.154

### Identification of Stably Expressed Reference Genes in Treated OPCs

According to *geNorm* algorithm, *Mrpl19* and *Hprt1* were identified in the microarray analysis as the best reference genes, followed by *B2m* (average *M*-value: 0.102 for *Mrpl19* and *Hprt1* genes and 0.147 for *B2m* gene). Surprisingly, *ActB* and *Gapdh*, mostly used as standard housekeeping gene for normalization, showed the most unstable gene expression in OPCs that were exposed to the CSF of our experimental conditions. The use of *NormFinder* software with the data showed that *Tfrc* and *B2m* were identified as the best reference genes followed by *ActB* (average *M*-value: 0.033 for *Tfrc* and *B2m* genes and 0.087 for *ActB* gene). *Mrpl19 and Gapdh* showed the least stable gene expression in OPCs that were exposed to the CSF of our experimental conditions. **Table 7** depicts candidate reference genes ranked in OPCs exposed to the CSF of RRMS and PPMS patients according to their expression stability by *geNorm* and *NormFinder* methods.

**Table 7:** Candidate reference genes ranked in OPCs exposed to the CSF of RRMS and PPMS patients according to their expression stability by *geNorm* and *NormFinder* methods.

*geNorm*			*NormFinder*		
Ranking Order	Gene name	Stability value (*M*)	Ranking order	Gene name	Stability value
1	*Mrpl19*	0.102	1	*Tfrc*	0.033
1	*Hprt1*	0.102	1	*B2m*	0.033
2	*B2m*	0.147	2	*ActB*	0.087
3	*Tfrc*	0.160	3	*Hprt1*	0.222
4	*ActB*	0.192	4	*Mrpl19*	0.260
5	*Gapdh*	0.272	5	*Gapdh*	0.426

*M: Average expression stability. Lower *M-*value indicates more stable expression while the highest *M-*value indicates variable expression.*

**FIGURE 1:**
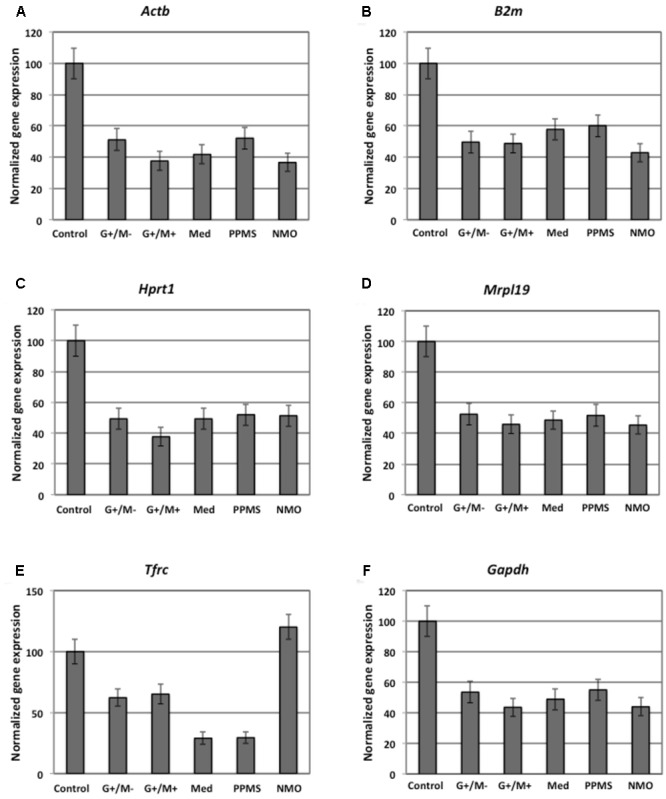
Normalized expression of **(A)** *ActB*; **(B)** *B2m*; **(C)** *Hprt*; **(D)** *Mrpl19*; **(E)** *Tfrc*; **(F)** *Gapdh* reference genes in OPCs tested in distinct disease courses of multiple sclerosis. G+/M- and G+/M+: Inflammatory forms of relapsing remitting multiple sclerosis; Med: Medullary form; PPMS: Primary progressive multiple sclerosis; NMO: Neuromyelitis Optica; Control: Other non-inflammatory neurological diseases (NIND).

The expression of β-actin (*ActB*) gene was downregulated significantly, showing 37 and 42% gene expression in OPCs exposed to the CSF of G+/M+ and medullary MS as compared to control. The expression was downregulated by 37% in OPCs exposed to the CSF of NMO patients as compared to control (**Figure 1A**). A marked fluctuation in *ActB* gene expression was seen in OPCs exposed to the CSF of various experimental conditions. *geNorm* also identified *ActB* as highly variable gene in these experimental conditions. We conclude that this gene is not suitable to normalize gene transcripts in treated OPCs.

The expression of β-2 microglobulin (*B2m*) gene was downregulated significantly showing 50, 48, and 58 % in OPCs exposed to the CSF of G+/M-, G+/M+ and medullary MS as compared to control. Similarly, significantly reduced expression with 43% in OPCs exposed to the CSF of NMO patients was observed as compared to control (**Figure 1B**). *geNorm* identified *B2m* as the third most stable gene hence this gene can be used for normalization purpose in treated OPCs.

The expression of hypoxanthine phosphoribosyltransferase (*Hprt1*) gene was downregulated significantly by 49, 38, and 49% in OPCs exposed to the CSF of G+/M-, G+/M+ and medullary MS as compared to control. Similarly the expression was around 52% significantly reduced in OPCs exposed to the CSF of PPMS and NMO patients as compared to control (**Figure 1C**). *geNorm* identified *Hprt1* as the most stable gene hence this gene can be used for normalization purpose in treated OPCs.

The expression of mitochondrial ribosomal protein L19 (*Mrpl19*) gene was downregulated significantly by 52, 46, and 48% in OPCs exposed to the CSF of G+/M-, G+/M+ and medullary MS as compared to control. Similarly the expression was 52 and 45% lower in OPCs exposed to the CSF of PPMS and NMO patients as compared to control (**Figure 1D**). *geNorm* identified *Mrpl19* as the most stable gene hence this gene can be used for normalization purpose in treated OPCs.

Similarly, reduced variation was observed in transferrin receptor (*Tfrc*) gene expression across all experimental conditions (**Figure 1E**).

The data indicates that the expression of glyceraldehyde 3-phosphate dehydrogenase (*Gapdh*) gene was downregulated by 44, 54, and 49% in OPCs treated with G+/M-, G+/M+ and medullary clinical form of MS as compared to OPCs exposed to the CSF of neurological controls. Similarly, the expression was reduced by 55 and 44% in OPCs treated with the CSF of PPMS and NMO patients as compared to OPCs exposed to the CSF of non-inflammatory neurological controls (NIND) (**Figure 1F**). According to *geNorm* and *NormFinder* algorithms, *Gapdh* was ranked as an unstable gene for normalizing mRNA transcripts.

We conclude from this data that *Mrpl19, Hprt, Tfrc*, and *B2m* should be used to normalize the gene transcripts in experiments related to the current one, without much differences between them, and the use of *ActB* and *Gapdh* should be avoided.

### Differential Expression of Genes Involved in Glucose Metabolism in OPCs by Microarray Gene Expression Profiling

**FIGURE 2:**
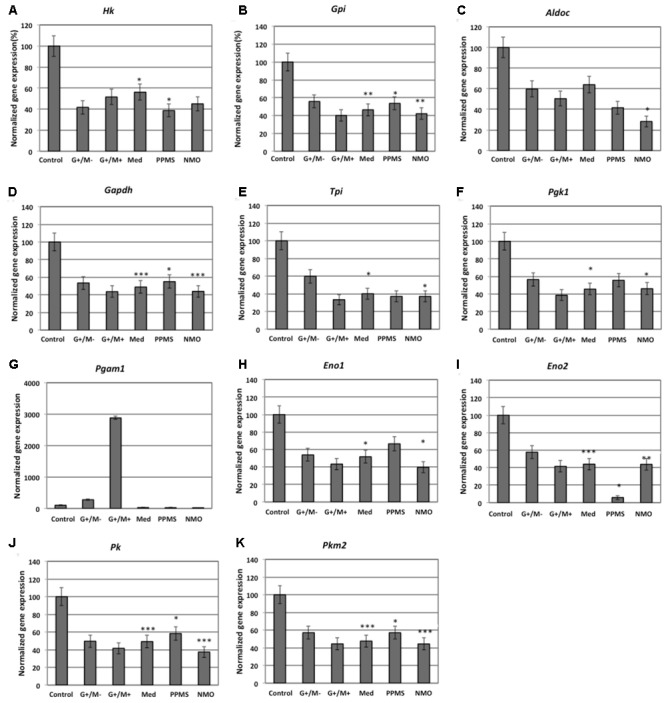
Normalized expression of **(A)** *Hk*; **(B)** *Gpi*; **(C)** *Aldoc*; **(D)** *Gapdh*; **(E)** *Tpi*; **(F)** *Pgk1*; **(G)** *Pgam1*; **(H)** *Eno1*; **(I)** *Eno2*; **(J)** *Pk*; **(K)** *Pkm2* genes involved in glycolytic pathway in OPCs treated with CSF of MS and NMO patients related to gene expression in OPCs treated with CSF of non-inflammatory neurological controls. OPCs: oligodendrocyte progenitor cells; Control: Neurons treated with CSF of non-inflammatory neurological controls; G+/M- and G+/M+: types of inflammatory MS; Med: Medullary MS; PP: Primary progressive multiple sclerosis; NMO: Neuromyelitis Optica. Four microarrays were used in each MS or NMO type of patients.

**FIGURE 3:**
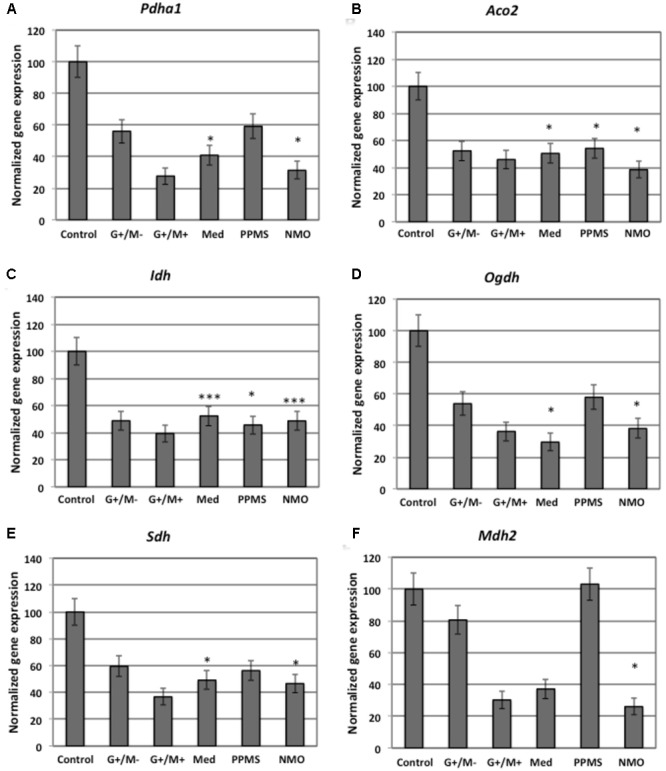
Normalized expression of **(A)** *Pdha1*; **(B)** *Aco2*; **(C)** *Idh*; **(D)** *Ogdh*; **(E)** *Sdh*; **(F)** *Mdh2* genes involved in TCA cycle in OPCs treated with CSF of MS and NMO patients related to gene expression in OPCs treated with CSF of non-inflammatory neurological controls. OPCs: oligodendrocyte progenitor cells; Control: Neurons treated with CSF of non-inflammatory neurological controls; G+/M- and G+/M+: types of inflammatory MS; Med: Medullary MS; PP: Primary progressive multiple sclerosis; NMO: Neuromyelitis Optica. Four microarrays were used in each MS or NMO type of patients.

**FIGURE 4:**
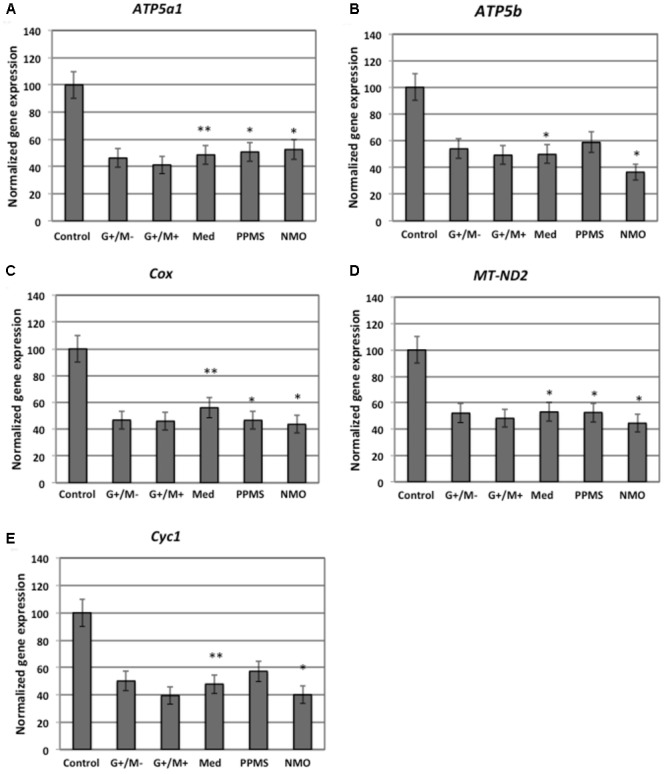
Normalized expression of **(A)** *ATP5a1*; **(B)** *ATP5b*; **(C)** *Cox*; **(D)** *MT-ND2*; **(E)** *Cyc1* genes involved in oxidative phosphorylation in OPCs treated with CSF of MS and NMO patients related to gene expression in OPCs treated with CSF of non-inflammatory neurological controls. OPCs: oligodendrocyte progenitor cells; Control: Neurons treated with CSF of non-inflammatory neurological controls; G+/M- and G+/M+: types of inflammatory MS; Med: Medullary MS; PP: Primary progressive multiple sclerosis; NMO: Neuromyelitis Optica. Four microarrays were used in each MS or NMO type of patients.

Our findings in the microarray analysis revealed that genes involved in carbohydrate metabolism were differentially expressed in our experimental conditions. **Figures 2–4** shows expression of genes involved in glycolytic pathway; TCA cycle; and oxidative phosphorylation in OPCs treated with CSF derived from MS and NMO patients normalized to gene expression in OPCs treated with CSF derived from non-inflammatory neurological controls. We have plotted the expression of genes in our experimental conditions, normalized with respect to neurological control, calculated from the absolute fold change values from microarray data.

When OPCs were exposed to CSF of G+/M- RRMS patients, the expression of several glycolytic genes including *Hk (hexokinase), Gpi (Glucose-6-phosphate isomerase), Gapdh (Glyceraldehyde 3-phosphate dehydrogenase), Tpi (Triosephosphate isomerase), Pgk1 (Phosphoglycerate kinase 1), Eno1 (Enolase 1), Eno2 (Enolase 2), Pk (Pyruvate kinase)*, and *Pkm2 (pyruvate kinase M2)* was found to be decreased (**Figures 2A,B,D–F,H–K**). Furthermore, genes implicated in TCA cycle including *Pdha1 (pyruvate dehydrogenase alpha 1), Aco2 (Aconitate hydratase), Idh (Isocitrate dehydrogenase)*, and *Ogdh (Oxoglutarate dehydrogenase)* were downregulated (**Figures 3A–D**). Similarly, genes involved in oxidative phosphorylation, namely *ATP5a1 (ATP synthase subunit alpha 1), ATP5b (ATP synthase subunit alpha 5b), MT-ND2 (mitochondrial encoded NADH dehydrogenase 2)*, and *Cyc1 (cytochrome c1)*, showed decreased expression as compared to neurological controls (**Figures 4A,B,D,E**).

When OPCs were exposed to CSF of G+/M+ RRMS patients, it was found that most of the enzymes involved in glycolysis including *Hk, Gpi, Gapdh, Tpi, Pgk1, Eno1*, and *Pk*, (**Figures 2A,B,D–H,J**); the related TCA cycle enzymes including *Pdha1, Aco2, Idh, Ogdh, Sdh (Succinate dehydrogenase)*, and *Mdh2 (Malate dehydrogenase 2)* (**Figures 3A–F**); and the mitochondrial electron chain enzymes [*MT-ND2, Cyc1, Cox (Cytochrome c oxidase), ATP5a1* and *ATP5b*] were strongly reduced in gene expression as compared to neurological controls (**Figures 4A–E**).

When OPCs were exposed to CSF of medullary patients, it was found that most of the enzymes involved in glycolysis including *Hk, Gpi, Gapdh, Tpi, Pgk1, Eno1, Eno2, Pk*, and *Pkm2* (**Figures 2A,B,D–F,H–K**), the related TCA cycle enzymes including *Pdha1, Aco2, Idh, Ogdh, Sdh*, and *Mdh2* (**Figures 3A–E**); and the mitochondrial electron chain enzymes (*MT-ND2, Cyc1, Cox, ATP5a1*, and *ATP5b*) were strongly reduced in gene expression as compared to neurological controls (**Figures 4A–E**).

In case of OPCs treated with CSF from PPMS patients, we found that most of the glycolytic genes, including *Hk1, Gpi, Gapdh, Tpi, Pgk1, Eno2, Pk*, and *Pkm2*, were reduced in gene expression (**Figures 2A,B,D–F,I–K**). Our findings also revealed down regulation of genes implicated in TCA cycle, including *Pdh, Aco2, Idh, Ogdh*, and *Sdh* (**Figures 3A–E**). Genes of ETC, including *ComplexI/NADH:ubiquinone oxidoreductase, Cyc1, Complex IV/COX* and *ATP synthase* (both alpha and beta subunits) showed down regulated gene expression (**Figures 4A–E**).

When OPCs were exposed to CSF of NMO patients, it was found that most of the enzymes involved in glycolysis including *Gpi, Aldoc (Fructose 1,6-bisphosphate aldolase), Gapdh, Tpi, Pgk1, Eno1, Pgam, Eno1, Eno2, Pk*, and *Pkm2* (**Figures 2B–F,H–K**); the related TCA cycle enzymes including *Pdha1, Aco2, Idh, Ogdh, Sdh*, and *Mdh2* (**Figures 3A–F**); and the mitochondrial electron chain enzymes (*MT-ND2, Cyc1, Cox, ATP5a1*, and *ATP5b*) were strongly reduced in gene expression as compared to neurological controls (**Figures 4A–E**).

Overall, the microarray data demonstrate that the genes involved in carbohydrate metabolism were differentially expressed in OPCs treated with the CSF from MS and NMO patients as compared to OPCs exposed to the CSF of neurological controls. We conclude that CSF exposure to OPCs altered the carbohydrate metabolism and may have altered the capacity of these cells to repair axonal damage in different clinical forms of MS.

### Analysis of Gene–Gene Interaction Networks Using String v10 Software

**FIGURE 5:**
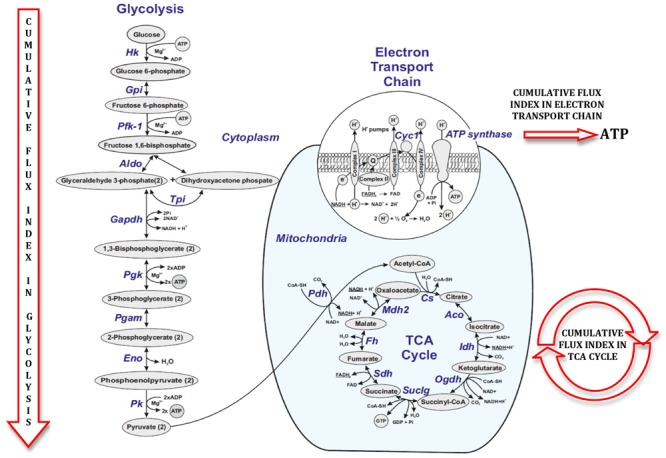
Figure illustrating cumulative flux index in glycolysis, TCA cycle and electron transport chain in glucose metabolic pathway.

**FIGURE 6:**
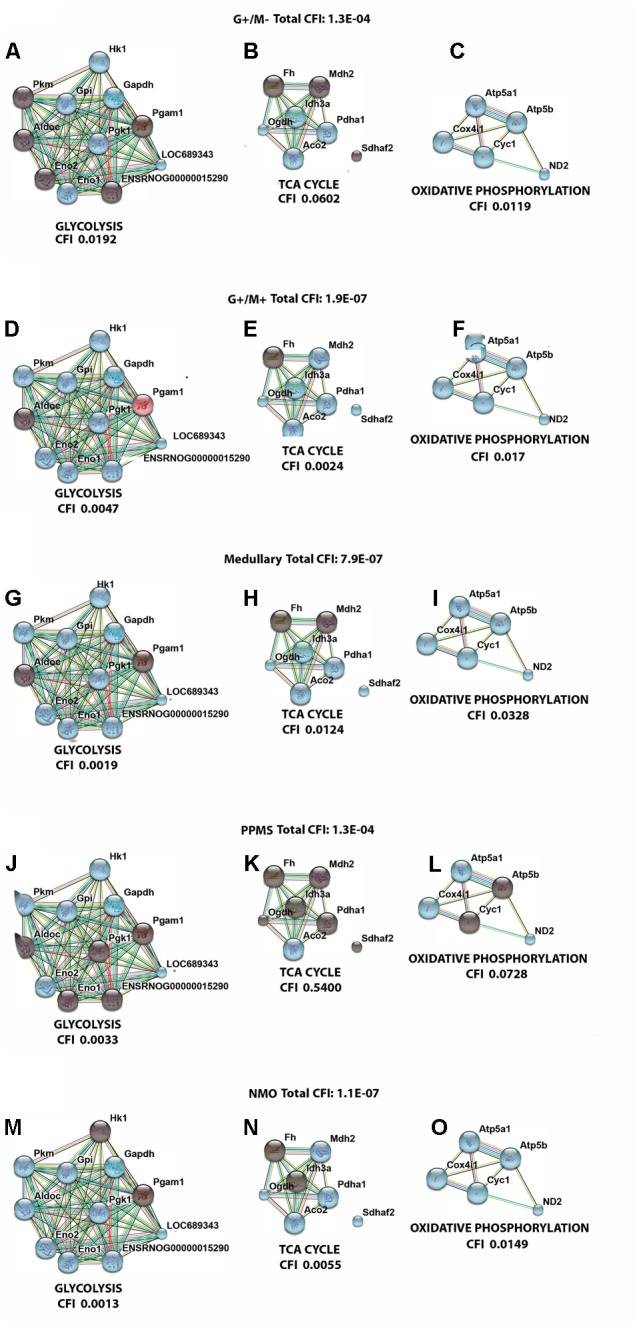
Visualization of gene interaction network generated by STRING v10 in OPCs exposed to CSF from **(A–C)** G+/M– RRMS; **(D–F)** G+/M+ RRMS; **(G–I)** Medullary MS; **(J–L)** PPMS; **(M–O)** NMO. Different line colors represent the types of evidence for the association between genes involved in **(A,D,G,J,M)** glycolysis; **(B,E,H,K,N)** TCA cycle and **(C,F,I,L,O)** oxidative phosphorylation in OPCs; Red color signifies upregulated expression, blue color signifies down regulated expression and gray color signifies genes that showed no variation in their expression in our experimental conditions. LOC689343 signifies *Pk* gene and ENSRNOG00000015290 signifies *Tpi* gene.

**Figure 5** illustrates a general metabolic network including glycolytic pathway, TCA cycle and electron transport chain with cumulative flux indexes. Gene–gene interaction network was visualized in OPCs exposed to CSF from G+/M- MS (**Figure 6A**); G+/M+ MS (**Figure 6B**); medullary MS (**Figure 6C**); PPMS (**Figure 6D**); and NMO patients (**Figure 6E**) generated by STRING v10. Significantly downregulated genes were indicated by blue color, and significantly upregulated genes were indicated by red color, in the STRING figure. The variation of metabolic flux, estimated with their values of CFI, in the different treatments with CSF of MS and NMO patients were integrated. CFI values were calculated as a parameter to integrate the reduced activity of the different enzymes in a specific network (glycolysis, TCA cycle and ATP generation, or together) as the expected total flux. This parameter is a simplified linear cumulative form of the flux control coefficients in the metabolic control analysis. This value roughly compares the different fluxes that may occur in the MS patients according to the number of enzymes down-regulated (after normalizing them by housekeeping gene expression) and the enzymatic activity level of each one. **Table 8** depicts upregulated and downregulated genes in distinct MS clinical forms and NMO and their related cumulative flux index values.

**Table 8:** Differentially expressed genes which are upregulated and downregulated in distinct MS clinical forms and NMO and their related cumulative flux index.

Clinical form	Genes upregulated			Genes downregulated			CFI			Total CFI	Prognosis
	Glycolysis	TCA cycle	ETC	Glycolysis	TCA cycle	ETC	Glycolysis	TCA cycle	ETC		
RRMS (G+/M-)	–	–	–	*Hk,Gpi, Gapdh, Pgk1, Eno, Pk*	*Pdh, Ogdh, Idh, Aco2*	*Atp5a1, Atp5b, Cyc1, Cox, MT-ND2*	0.0192	0.0602	0.0119	1.3E-04	Poor prognosis
RRMS (G+/M+)	*Pgam*	–	–	*Hk, Gpi, Gapdh,TpiPgk1, Eno, Pk*	*Pdh, Mdh2,Sdh, Ogdh, Idh, Aco2*	*Atp5a1, Atp5b, Cyc1, Cox, MT-ND2*	0.0047	0.0024	0.017	1.9E-07	Worst prognosis
Med	–	–	–	*Hk, Gpi, Gapdh, Tpi, Pgk1, Eno, Pk*	*Pdh, Sdh, Ogdh, Idh, Aco2*	*Atp5a1, Atp5b, Cyc1, Cox, MT-ND2*	0.0019	0.0124	0.0328	7.9E-07	Worst prognosis
PPMS	–	–	–	*Hk, Gpi, Gapdh, Eno2, Pk*	*Aco2*	*Atp5a1, Cox, MT-ND2*	0.0033	0.5400	0.0728	1.3E-04	–
NMO	–	–	–	*Gpi, Aldoc Gapdh, Tpi, Pgk1, Eno, Pk*	*Pdh, Mdh2, Sdhaf2, Ogdh, Aco2*	*Atp5a1, Atp5b, Cyc1, Cox, MT-ND2*	0.0013	0.0055	0.0149	1.1E-07	–

With the calculation of CFI parameters, we may say that whole carbohydrate metabolic flux and ATP synthesis decreased in OPCs when exposed to CSF derived from MS and NMO patients. Our findings suggest a significant downregulation of genes involved in carbohydrate metabolism suggesting that factors present in the CSF, in our model, perturb the metabolism of OPCs.

## Discussion

In the present study, we found a downregulated expression of genes involved in carbohydrate metabolism in OPCs exposed to CSF from MS and NMO patients as compared to controls. We found that total cumulative flux index associated with glycolysis, TCA cycle and ATP generation declined to a great extent in G+/M+ RRMS patients (CFI: 1.9E-07) as compared to G+/M- RRMS subtype (CFI: 1.3E-04), where lower energy level may have reduced the repair process by the OPCs. G+/M- RRMS is the less severe and less aggressive form displaying oligoclonal bands of IgG antibodies (+) but no IgM (-) oligoclonal antibodies in the CSF of brain. On the other hand, G+/M+ clinical form of RRMS displayed oligoclonal bands of both IgG antibodies (+) and IgM (+) antibodies in the CSF of brain. This form was more aggressive with worse prognosis than G+/M- RRMS. In G+/M- patients, metabolic genes showed a downregulated expression leading to a reduction in the overall metabolic flux. This caused a decrease in ATP synthesis and overall ability of OPCs to repair the damaged neuronal cells, which can be related to poor prognosis and development of pathology in these patients. Our results are consistent with previous findings, which showed a reduction in ATP synthase expression in MS lesions (Smith and Lassmann, 2002). It may be indicated that genes involved in metabolism of carbohydrates and ATP synthesis were strongly affected in G+/M+ subtype in OPCs as compared to G+/M- subtype. The energy required to repair damaged neurons degenerated in MS lesions could be much lower in G+/M- RRMS less severe clinical form as compared to more aggressive G+/M+ RRMS. To combat oxidative stress generated in neurological diseases, the cells need to produce large quantities of reducing equivalents for energy production. However, insufficient energy production severely impaired the ability to repair nerve damage. We conclude that the differential expression of metabolic genes reduced the repairing potential by OPCs which could be related to worse prognosis in patients with G+/M+ RRMS type as compared to G+/M- RRMS.

In medullary MS, our results showed a significant reduction of several metabolic genes as compared to OPCs exposed to the CSF from neurological controls. Compared with non-MS patients (controls), total cumulative flux index in medullary MS dropped to a very low value (CFI: 7.9E-07) in all three metabolic pathways, similar to what occurred in G+/M+ RRMS. The most aggressive form was classified as medullary MS with a predominant affectation of the spinal cord. All these patients were positive for oligoclonal IgG bands (OCGB) and negative for oligoclonal IgM bands (OCMB) in CSF of spinal region. The drastic reduction of metabolic genes expression in both G+/M+ RRMS and medullary MS would result in bioenergetic failure eventually causing OPCs to reduce the repair of axonal damage which is correlated with worst prognosis compared with G+/M- RRMS.

Moreover, our findings demonstrated a reduced expression of genes involved in glucose metabolism in OPCs treated with CSF from PPMS patients. Altogether, total cumulative flux index in carbohydrates and ATP production was decreased strongly in OPCs (CFI: 1.3E-04) in this subtype of patients. Relapsing remitting MS (RRMS) is the most common form of MS affecting 80–90% of the patients. On the contrary, patients with primary progressive MS (PPMS) are characterized by a steady worsening of neurological symptoms with no relapse or remission affecting 10–15% of the patients. Our findings are consistent with the fact that PPMS patients are less aggressive, slower and have progressive course and a better prognosis than RRMS subtypes G+/M+ or medullary MS.

Finally, we observed a reduced carbohydrate metabolism and ATP synthesis in OPCs exposed to CSF of NMO patients compared to non-MS (controls). In addition, we found a decrease in the cumulative flux index accumulated in all three pathways.

The results indicate that the flux indexes accumulated in OPCs exposed to CSF of NMO patients decreased significantly (CFI: 1.1E-07). It has been documented that anti-NMO IgG does not have a direct effect over OPCs, and it has high affinity for the astroglial receptors (Marignier et al. 2010). Despite purified cultures of OPCs were used, the 1% presence of astrocytes could explain, although unlikely, the downregulation of metabolic genes in OPCs treated with NMO derived CSF. Another, more plausible explanation (not tested), is that some NMO patients could have presented anti-MOG, a recent antibody against myelin associated oligodendrocyte, that in cases of NMO patients caused a profound oligodendropathy non-associated to astrocyte damage (Ikeda et al., 2015). Because anti-MOG is present in 40% of seronegative-NMO, and in our series we had four NMO patients with no anti-NMO antibodies, we cannot rule out the possibility that some patients presented anti-MOG.

Several studies have demonstrated the unmet need of energy requirement in MS, which include mitochondrial impairment in cultured neurons (Kim et al., 2010), animal models of MS (Nikić et al., 2011) and in MS samples (Dutta et al., 2006). Metabolic abnormalities are implicated in the pathogenesis of neurodegenerative diseases (Henneman et al., 1954; McArdle et al., 1960; Lu et al., 2000; Dutta et al., 2006). In MS literature indicating any association between perturbed glucose metabolism with its pathogenesis is meager. Royds et al. (1981), observed an increased activity of metabolic enzymes including enolase, pyruvate kinase, lactate dehydrogenase and aldolase in the CSF of patients with disseminated sclerosis. Kölln et al. (2006) demonstrated that B cells and antibodies reactive with *Tpi* and *Gapdh* are produced intrathecally in CSF and lesions of MS. Both TPI and GAPDH are essential metabolic enzymes involved in ATP production. Another investigation by the same group showed that these antibodies bind with TPI and GADPH, and inhibit the glycolytic activity of GAPDH but not TPI in MS patients (Kölln et al., 2010).

The main findings of this study revealed a disturbed carbohydrate metabolism in OPCs treated with the CSF derived from MS and NMO subjects. Factors present in the CSF, in our model, affected the metabolism of OPCs and clearly differentiate more benign forms from the most aggressive forms in MS. The effect of CSF was different in MS aggressivity of RRMS and PPMS clinical form (G+/M-, G+/M+, Med, PPMS). G+/M+ RRMS and medullary derived CSF treated OPCs were strongly affected by reducing carbohydrate metabolism as evidenced by down regulation of most of the genes which is suggestive of least ATP synthesis. This indicates blockage of myelin repair by OPCs and correlated with worst prognosis. OPCs treated with CSF from G+/M- RRMS patients demonstrates slightly reduced carbohydrate metabolism correlated with poor prognosis.

Finally, our *geNorm* analysis revealed *Mrpl19* and *Hprt* as the most stably expressed genes followed by *B2m*, whereas β-*actin* and *Gapdh* were least stably expressed genes when OPCs were treated with diseased CSF, as revealed by *geNorm* analysis. Our findings are consistent with previous findings in which *Gapdh* and β*-actin* have been demonstrated to show variable expression in different experimental conditions (Hamalainen et al., 2001; Deindl et al., 2002; Glare et al., 2002; Radonic et al., 2004; Mathur et al., 2015). The results allowed us to differentiate different clinical forms and aggressivity in MS and MS from NMO. However, it is still elusive whether these alterations in metabolic gene expression cause MS and NMO or are a consequence of the disease. These findings open new avenues of study and allow the development of therapeutic agents targeted to restore the metabolic function and hence repair and/or prevent axonal damage responsible for functional disability in the patient. A greater understanding of these impaired metabolic pathways may offer new insights into more efficacious treatments for MS and NMO.

## Ethics Statement

All subjects gave written informed consent in accordance with the Declaration of Helsinki. The study protocol was approved by the Institutional Ethical Committee.

## Author Contributions

DM conducted experiments, analyzed and wrote the manuscript. AR-C, JC, JH, OV, and FZ conducted experiments. FC-F and PC designed the study. BC and GL-R designed the study and edited and revised the manuscript.

## Conflict of Interest Statement

The authors declare that the research was conducted in the absence of any commercial or financial relationships that could be construed as a potential conflict of interest.

## Footnotes

1 http://www.bioconductor.org/

2 http://cran.r-project.org/

3 http://string-db.org

## References

[B1] Afifi A. K. Aleu F. P. Goodgold J. MacKay B. (1966). Ultrastructure of atrophic muscle in amyotrophic lateral sclerosis. *Neurology* 16 475–481. 10.1212/WNL.16.5.475 5949060

[B2] Alcázar A. Regidor I. Masjuan J. Salinas M. Alvarez-Cermeño J. C. (2000). Axonal damage induced by cerebrospinal fluid from patients with relapsing-remitting multiple sclerosis. *J. Neuroimmunol.* 104 58–67. 10.1016/S0165-5728(99)00225-8 10683515

[B3] Atsumi T. (1981). The ultrastructure of intramuscular nerves in amyotrophic lateral sclerosis. *Acta Neuropathol.* 55 193–198. 10.1007/BF00691318 7349578

[B4] Becker K. G. Mattson D. H. Powers J. M. Gado A. M. Biddison W. E. (1997). Analysis of a sequenced cDNA library from multiple sclerosis lesions. *J. Neuroimmunol.* 77 27–38. 10.1016/S0165-5728(97)00045-3 9209265

[B5] Blalock E. M. Geddes J. W. Chen K. C. Porter N. M. Markesbery W. R. Landfield P. W. (2004). Incipient Alzheimer’s disease: microarray correlation analyses reveal major transcriptional and tumor suppressor responses. *Proc. Natl. Acad. Sci. U.S.A.* 101 2173–2178. 10.1073/pnas.0308512100 14769913 PMC357071

[B6] Bomprezzi R. Ringnér M. Kim S. Bittner M. L. Khan J. Chen Y. (2003). Gene expression profile in multiple sclerosis patients and healthy controls: identifying pathways relevant to disease. *Hum. Mol. Genet.* 12 2191–2199. 10.1093/hmg/ddg221 12915464

[B7] Borthwick G. M. Johnson M. A. Ince P. G. Shaw P. J. Turnbull D. M. (1999). Mitochondrial enzyme activity in amyotrophic lateral sclerosis: implications for the role of mitochondria in neuronal cell death. *Ann. Neurol.* 46 787–790. 10.1002/1531-8249(199911)46:5<787::AID-ANA17>3.0.CO;2-8 10553999

[B8] Bowling A. C. Schulz J. B. Brown R. H. Jr. Beal M. F. (1993). Superoxide dismutase activity, oxidative damage, and mitochondrial energy metabolism in familial and sporadic amyotrophic lateral sclerosis. *J. Neurochem.* 61 2322–2325. 10.1111/j.1471-4159.1993.tb07478.x 8245985

[B9] Broadwater L. Pandit A. Clements R. Azzam S. Vadnal J. Sulak M. (2011). Analysis of the mitochondrial proteome in multiple sclerosis cortex. *Biochim. Biophys. Acta* 1812 630–641. 10.1016/j.bbadis.2011.01.012 21295140 PMC3074931

[B10] Brooks W. M. Lynch P. J. Ingle C. C. Hatton A. Emson P. C. Faull R. L. (2007). Gene expression profiles of metabolic enzyme transcripts in Alzheimer’s disease. *Brain Res.* 1127 127–135. 10.1016/j.brainres.2006.09.106 17109828

[B11] Browne S. E. Yang L. DiMauro J. P. Fuller S. W. Licata S. C. Beal M. F. (2006). Bioenergetic abnormalities in discrete cerebral motor pathways presage spinal cord pathology in the G93A SOD1 mouse model of ALS. *Neurobiol. Dis.* 22 599–610. 10.1016/j.nbd.2006.01.001 16616851

[B12] Brynedal B. Khademi M. Wallström E. Hillert J. Olsson T. Duvefelt K. (2010). Gene expression profiling in multiple sclerosis: a disease of the central nervous system, but with relapses triggered in the periphery? *Neurobiol. Dis.* 37 613–621. 10.1016/j.nbd.2009.11.014 19944761

[B13] Chabas D. Baranzini S. E. Mitchell D. Bernard C. C. Rittling S. R. Denhardt D. T. (2001). The influence of the proinflammatory cytokine, osteopontin, on autoimmune demyelinating disease. *Science* 294 1731–1735. 10.1126/science.1062960 11721059

[B14] Dal Canto M. C. Gurney M. E. (1995). Neuropathological changes in two lines of mice carrying a transgene for mutant human Cu, Zn SOD, and in mice overexpressing wild type human SOD: a model of familial amyotrophic lateral sclerosis (FALS). *Brain Res.* 676 25–40. 10.1016/0006-8993(95)00063-V 7796176

[B15] Dalakas M. C. Hatazawa J. Brooks R. A. Di Chiro G. (1987). Lowered cerebral glucose utilization in amyotrophic lateral sclerosis. *Ann. Neurol.* 22 580–586. 10.1002/ana.410220504 3501273

[B16] Deindl E. Boengler K. van Royen N. Schaper W. (2002). Differential expression of GAPDH and beta3-actin in growing collateral arteries. *Mol. Cell. Biochem.* 236 139–146. 10.1023/A:1016166127465 12190113

[B17] Der S. D. Zhou A. Williams B. R. G. Silverman R. H. (1998). Identification of genes differentially regulated by interferon α, β, or γ using oligonucleotide arrays. *Proc. Natl. Acad. Sci. U.S.A.* 95 15623–15628. 10.1073/pnas.95.26.15623 9861020 PMC28094

[B18] Dodge J. C. Treleaven C. M. Fidler J. A. Tamsett T. J. Bao C. Searles M. (2013). Metabolic signatures of amyotrophic lateral sclerosis reveal insights into disease pathogenesis. *Proc. Natl. Acad. Sci. U.S.A.* 110 10812–10817. 10.1073/pnas.1308421110 23754387 PMC3696768

[B19] Dutta R. McDonough J. Yin X. Peterson J. Chang A. Torres T. (2006). Mitochondrial dysfunction as a cause of axonal degeneration in multiple sclerosis patients. *Ann. Neurol.* 59 478–489. 10.1002/ana.20736 16392116

[B20] Echaniz-Laguna A. Zoll J. Ponsot E. N’guessan B. Tranchant C. Loeffler J. P. (2006). Muscular mitochondrial function in amyotrophic lateral sclerosis is progressively altered as the disease develops: a temporal study in man. *Exp. Neurol.* 198 25–30. 10.1016/j.expneurol.2005.07.020 16126198

[B21] Everaert B. R. Boulet G. A. Timmermans J. P. Vrints C. J. (2011). Importance of suitable reference gene selection for quantitative real-time PCR: special reference to mouse myocardial infarction studies. *PLoS ONE* 6:e23793 10.1371/journal.pone.0023793 PMC3157472 21858224

[B22] Fort P. Marty L. Piechaczyk M. el Sabrouty S. Dani C. Jeanteur P. (1985). Various rat adult tissues express only one major mRNA species from the glyceraldehyde-3-phosphate-dehydrogenase multigenic family. *Nucleic Acids Res.* 13 1431–1442. 10.1093/nar/13.5.1431 2987824 PMC341087

[B23] Fujita K. Yamauchi M. Shibayama K. Ando M. Honda M. Nagata Y. (1996). Decreased cytochrome *c* oxidase activity but unchanged superoxide dismutase and glutathione peroxidase activities in the spinal cords of patients with amyotrophic lateral sclerosis. *J. Neurosci. Res.* 45 276–281. 10.1002/(SICI)1097-4547(19960801)45:3<276::AID-JNR9>3.0.CO;2-A 8841988

[B24] Glare E. M. Divjak M. Bailey M. J. Walters E. H. (2002). β-Actin and GAPDH housekeeping gene expression in asthmatic airways is variable and not suitable for normalising mRNA levels. *Thorax* 57 765–770. 10.1136/thorax.57.9.765 12200519 PMC1746418

[B25] Gonzalez de Aguilar J.-L. Dupuis L. Oudart H. Loeffler J.-P. (2005). The metabolic hypothesis in amyotrophic lateral sclerosis: insights from mutant Cu/Zn-superoxide dismutase mice. *Biomed. Pharmacother.* 59 190–196. 10.1016/j.biopha.2005.03.003 15862714

[B26] Gorzelniak K. Janke J. Engeli S. Sharma A. M. (2001). Validation of endogenous controls for gene expression studies in human adipocytes and preadipocytes. *Horm. Metab. Res.* 33 625–627. 10.1055/s-2001-17911 11607884

[B27] Gubern C. Hurtado O. Rodrguez R. Morales J. R. Romera V. G. Moro M. A. (2009). Validation of housekeeping genes for quantitative real-time PCR in in-vivo and in-vitro models of cerebral ischaemia. *BMC Mol. Biol.* 10:57 10.1186/1471-2199-10-57 PMC2706836 19531214

[B28] Haines J. D. Vidaurre O. G. Zhang F. Riffo-Campos ÁL. Castillo J. Casanova B. (2015). Multiple sclerosis patient-derived CSF induces transcriptional changes in proliferating oligodendrocyte progenitors. *Mult. Scler. J.* 21 1655–1669. 10.1177/1352458515573094 PMC4628566 25948622

[B29] Hamalainen H. K. Tubman J. C. Vikman S. Kyrola T. Ylikoski E. Warrington J. A. (2001). Identification and validation of endogenous reference genes for expression profiling of T helper cell differentiation by quantitative real-time RT-PCR. *Anal. Biochem.* 299 63–70. 10.1006/abio.2001.5369 11726185

[B30] Harrison D. C. Medhurst A. D. Bond B. C. Campbell C. A. Davis R. P. Philpott K. L. (2000). The use of quantitative RT-PCR to measure mRNA expression in a rat model of focal ischemia – caspase-3 as a case study. *Brain Res. Mol. Brain Res.* 75 143–149. 10.1016/S0169-328X(99)00305-8 10648898

[B31] Henneman D. H. Altschule M. D. Goncz R. M. Alexander L. (1954). Carbohydrate metabolism in brain disease. I. Glucose metabolism in multiple sclerosis. *AMA Arch. Neurol. Psychiatry* 72 688–695. 10.1001/archneurpsyc.1954.02330060024004 13206487

[B32] Hong J. Zang Y. C. Hutton G. Rivera V. M. Zhang J. Z. (2004). Gene expression profiling of relevant biomarkers for treatment evaluation in multiple sclerosis. *J. Neuroimmunol.* 152 126–139. 10.1016/j.jneuroim.2004.03.004 15223245

[B33] Ikeda K. Kiyota N. Kuroda H. Sato D. K. Nishiyama S. Takahashi T. (2015). Severe demyelination but no astrocytopathy in clinically definite neuromyelitis optica with anti-myelin-oligodendrocyte glycoprotein antibody. *Mult. Scler.* 21 656–659. 10.1177/1352458514551455 25257613

[B34] Iñarrea P. Alarcia R. Alava M. A. Capablo J. L. Casanova A. Iñiguez C. (2013). Mitochondrial Complex Enzyme Activities and Cytochrome c Expression Changes in Multiple Sclerosis. *Mol. Neurobiol.* 49 1–9. 10.1007/s12035-013-8481-z 23761047

[B35] Jones H. H. Jones H. H. Jr. Bunch L. D. (1950). Biochemical studies in multiple sclerosis. *Ann. Intern. Med.* 33 831–840. 10.7326/0003-4819-33-4-831 14771754

[B36] Jung C. Higgins C. M. J. Xu Z. (2002). Mitochondrial electron transport chain complex dysfunction in a transgenic mouse model for amyotrophic lateral sclerosis. *J. Neurochem.* 83 535–545. 10.1046/j.1471-4159.2002.01112.x 12390515

[B37] Kim J. Y. Shen S. Dietz K. He Y. Howell O. Reynolds R. (2010). HDAC1 nuclear export induced by pathological conditions is essential for the onset of axonal damage. *Nat. Neurosci.* 13 180–189. 10.1038/nn.2471 20037577 PMC2829989

[B38] Koike F. Satoh J. Miyake S. Yamamoto T. Kawai M. Kikuchi S. (2003). Microarray analysis identifies interferon beta-regulated genes in multiple sclerosis. *J. Neuroimmunol.* 139 109–118. 10.1016/S0165-5728(03)00155-3 12799028

[B39] Kölln J. Ren H. M. Da R. R. Zhang Y. Spillner E. Olek M. (2006). Triosephosphate isomerase- and glyceraldehyde-3-phosphate dehydrogenase-reactive autoantibodies in the cerebrospinal fluid of patients with multiple sclerosis. *J. Immunol.* 177 5652–5658. 10.4049/jimmunol.177.8.5652 17015754

[B40] Kölln J. Zhang Y. Thai G. Demetriou M. Hermanowicz N. Duquette P. (2010). Inhibition of glyceraldehyde-3-phosphate dehydrogenase activity by antibodies present in the cerebrospinal fluid of patients with multiple sclerosis. *J. Immunol.* 185 1968–1975. 10.4049/jimmunol.0904083 20610654

[B41] Kong J. Xu Z. (1998). Massive mitochondrial degeneration in motor neurons triggers the onset of amyotrophic lateral sclerosis in mice expressing a mutant SOD1. *J. Neurosci.* 18 3241–3250. 9547233 10.1523/JNEUROSCI.18-09-03241.1998 PMC6792665

[B42] Kostulas V. K. Link H. Lefvert A. K. (1987). Oligoclonal IgG bands in cerebrospinal fluid. Principles for demonstration and interpretation based on findings in 1114 neurological patients. *Arch. Neurol.* 44 1041–1044. 10.1001/archneur.1987.00520220043014 3632376

[B43] Lee Y. Morrison B. M. Li Y. Lengacher S. Farah M. H. Hoffman P. N. (2012). Oligodendroglia metabolically support axons and contribute to neurodegeneration. *Nature* 487 443–448. 10.1038/nature11314 22801498 PMC3408792

[B44] Lin J. Diamanduros A. Chowdhury S. A. Scelsa S. Latov N. Sadiq S. A. (2009). Specific electron transport chain abnormalities in amyotrophic lateral sclerosis. *J. Neurol.* 256 774–782. 10.1007/s00415-009-5015-8 19240958

[B45] Lu F. Selak M. O’Connor J. Croul S. Lorenzana C. Butunoi C. (2000). Oxidative damage to mitochondrial DNA and activity of mitochondrial enzymes in chronic active lesions of multiple sclerosis. *J. Neurol. Sci.* 177 95–103. 10.1016/S0022-510X(00)00343-9 10980305

[B46] Lublin F. D. Reingold S. C. (1996). Defining the clinical course of multiple sclerosis: results of an international survey. National Multiple Sclerosis Society (USA) Advisory Committee on Clinical Trials of New Agents in Multiple Sclerosis. *Neurology* 46 907–911. 10.1212/WNL.46.4.907 8780061

[B47] Mahad D. Ziabreva I. Lassmann H. Turnbull D. (2008). Mitochondrial defects in acute multiple sclerosis lesions. *Brain* 131 1722–1735. 10.1093/brain/awn105 18515320 PMC2442422

[B48] Marignier R. Nicolle A. Watrin C. Touret M. Cavagna S. Varrin-Doyer M. (2010). Oligodendrocytes are damaged by neuromyelitis optica immunoglobulin G via astrocyte injury. *Brain* 133 2578–2591. 10.1093/brain/awq177 20688809

[B49] Mathur D. López-Rodas G. Casanova B. Marti M. B. (2014). Perturbed glucose metabolism: insights into multiple sclerosis pathogenesis. *Front. Neurol.* 5:250 10.3389/fneur.2014.00250 PMC4249254 25520698

[B50] Mathur D. Urena-Peralta J. R. Lopez-Rodas G. Casanova B. Coret-Ferrer F. Burgal-Marti M. (2015). Bypassing hazard of housekeeping genes: their evaluation in rat granule neurons treated with cerebrospinal fluid of multiple sclerosis subjects. *Front. Cell. Neurosci.* 9:375 10.3389/fncel.2015.00375 PMC4585208 26441545

[B51] Mattiazzi M. D’Aurelio M. Gajewski C. D. Martushova K. Kiaei M. Beal M. F. (2002). Mutated human SOD1 causes dysfunction of oxidative phosphorylation in mitochondria of transgenic mice. *J. Biol. Chem.* 277 29626–29633. 10.1074/jbc.M203065200 12050154

[B52] Mazzola J. L. Sirover M. A. (2001). Reduction of glyceraldehyde-3-phosphate dehydrogenase activity in Alzheimer’s disease and in Huntington’s disease fibroblasts. *J. Neurochem.* 76 442–449. 10.1046/j.1471-4159.2001.00033.x 11208907

[B53] McArdle B. Mackenzie I. C. K. Webster G. R. (1960). Studies on intermediate carbohydrate metabolism in Multiple Sclerosis. *J. Neurol. Neurosurg. Psychiatry* 23 127–132. 10.1136/jnnp.23.2.127 21610891 PMC495342

[B54] McCarthy K. D. de Vellis J. (1980). Preparation of separate astroglial and oligodendroglial cell cultures from rat cerebral tissue. *J. Cell Biol.* 85 890–902. 10.1083/jcb.85.3.890 6248568 PMC2111442

[B55] Medhurst A. D. Harrison D. C. Read S. J. Campbell C. A. Robbins M. J. Pangalos M. N. (2000). The use of TaqMan RT-PCR assays for semiquantitative analysis of gene expression in CNS tissues and disease models. *J. Neurosci. Methods* 98 9–20. 10.1016/S0165-0270(00)00178-3 10837866

[B56] Nelissen K. Smeets K. Mulder M. Hendriks J. Ameloot M. (2010). Selection of reference genes for gene expression studies in rat oligodendrocytes using quantitative real time PCR. *J. Neurosci. Methods* 187 78–83. 10.1016/j.jneumeth.2009.12.018 20036692

[B57] Nikić I. Merkler D. Sorbara C. Brinkoetter M. Kreutzfeldt M. Bareyre F. M. (2011). A reversible form of axon damage in experimental autoimmune encephalomyelitis and multiple sclerosis. *Nat. Med.* 17 495–499. 10.1038/nm.2324 21441916

[B58] Ohl F. Jung M. Xu C. Stephan C. Rabien A. Burkhardt M. (2005). Gene expression studies in prostate cancer tissue: Which reference gene should be selected for normalization? *J. Mol. Med.* 83 1014–1024. 10.1007/s00109-005-0703-z 16211407

[B59] Radonic A. Thulke S. Mackay I. M. Landt O. Siegert W. Nitsche A. (2004). Guideline to reference gene selection for quantitative real-time PCR. *Biochem. Biophys. Res. Commun.* 313 856–862. 10.1016/j.bbrc.2003.11.177 14706621

[B60] Royds J. A. Timperley W. R. Taylor C. B. (1981). Levels of enolase and other enzymes in the cerebrospinal fluid as indices of pathological change. *J. Neurol. Neurosurg. Psychiatry* 44 1129–1135. 10.1136/jnnp.44.12.1129 7334408 PMC491233

[B61] Safavizadeh N. Rahmani S. A. Zaefizadeh M. (2013). Investigation of cytocrom c oxidase gene subunits expression on the Multiple sclerosis. *Indian J. Hum. Genet.* 19 18–25. 10.4103/0971-6866.112879 23901189 PMC3722625

[B62] Sasaki S. Iwata M. (1996). Ultrastructural study of synapses in the anterior horn neurons of patients with amyotrophic lateral sclerosis. *Neurosci. Lett.* 204 53–56. 10.1016/0304-3940(96)12314-4 8929976

[B63] Senatorov V. V. Charles V. Reddy P. H. Tagle D. A. Chuang D. M. (2003). Overexpression and nuclear accumulation of glyceraldehyde-3- phosphate dehydrogenase in a transgenic mouse model of Huntington’s disease. *Mol. Cell. Neurosci.* 22 285–297. 10.1016/S1044-7431(02)00013-1 12691731

[B64] Sharief M. K. Thompson E. J. (1991). Intrathecal immunoglobulin M synthesis in multiple sclerosis. *Brain* 114 181–195. 1998881

[B65] Siklós L. Engelhardt J. Harati Y. Smith R. G. Joó F. Appel S. H. (1996). Ultrastructural evidence for altered calcium in motor nerve terminals in amyotropic lateral sclerosis. *Ann. Neurol.* 39 203–216. 10.1002/ana.410390210 8967752

[B66] Smith K. J. Lassmann H. (2002). The role of nitric oxide in multiple sclerosis. *Lancet Neurol.* 1 232–241. 10.1016/S1474-4422(02)00102-3 12849456

[B67] Smyth G. K. (2004). Linear models and empirical Bayes methods for assessing differential expression in microarray experiments. *Stat. Appl. Genet. Mol. Biol.* 3:3 10.2202/1544-6115.1027 16646809

[B68] Soucek T. Cumming R. Dargusch R. Maher P. Schubert D. (2003). The regulation of glucose metabolism by HIF-1 mediates a neuroprotective response to amyloid beta peptide. *Neuron* 39 43–56. 10.1016/S0896-6273(03)00367-2 12848931

[B69] Stürzenbaum S. R. Kille P. (2001). Control genes in quantitative molecular biological techniques: the variability of invariance. *Comp. Biochem. Physiol. B Biochem. Mol. Biol.* 130 281–289. 10.1016/S1096-4959(01)00440-7 11567890

[B70] Szklarczyk D. Franceschini A. Wyder S. Forslund K. Heller D. Huerta-Cepas J. (2015). STRING v10: protein–protein interaction networks, integrated over the tree of life. *Nucleic Acids Res.* 43 D447–D452. 10.1093/nar/gku1003 25352553 PMC4383874

[B71] Toegel S. Huang W. Piana C. Unger F. M. Wirth M. Goldring M. B. (2007). Selection of reliable reference genes for qPCR studies on chondroprotective action. *BMC Mol. Biol.* 8:13 10.1186/1471-2199-8-13 PMC1820791 17324259

[B72] Torres J. M. Gómez-Capilla J. A. Ruiz E. Ortega E. (2003). Semiquantitative RT-PCR method coupled to capillary electrophoresis to study 5α-reductase mRNA isozymes in rat ventral prostate in different androgen status. *Mol. Cell. Biochem.* 250 125–130. 10.1023/A:1024902419502 12962150

[B73] Tricarico C. Pinzani P. Bianchi S. Paglierani M. Distante V. Pazzagli M. (2002). Quantitative real-time reverse transcription polymerase chain reaction: normalization to rRNA or single housekeeping genes is inappropriate for human tissue biopsies. *Anal. Biochem.* 309 293–300. 10.1016/S0003-2697(02)00311-1 12413463

[B74] Vandesompele J. De Preter K Pattyn F. Poppe B. Van Roy N De Paepe A (2002). Accurate normalization of real-time quantitative RT-PCR data by geometric averaging of multiple internal control genes. *Genome Biol.* 3:RESEARCH0034 10.1186/gb-2002-3-7-research0034 PMC126239 12184808

[B75] Vidaurre O. G. Haines J. D. Katz Sand I Adula K. P. Huynh J. L. McGraw C. A. (2014). Cerebrospinal fluid ceramides from patients with multiple sclerosis impair neuronal bioenergetics. *Brain* 137(Pt 8) 2271–2286. 10.1093/brain/awu139 24893707 PMC4164163

[B76] Villar L. M. Casanova B. Ouamara N. Comabella M. Jalili F. Leppert D. (2014). Immunoglobulin M oligoclonal bands: biomarker of targetable inflammation in primary progressive multiple sclerosis. *Ann. Neurol.* 76 231–240. 10.1002/ana.24190 24909126

[B77] Wandinger K. P. Stürzebecher C. S. Bielekova B. Detore G. Rosenwald A. Staudt L. M. (2001). Complex immunomodulatory effects of interferon-beta in multiple sclerosis include the upregulation of T helper 1-associated marker genes. *Ann. Neurol.* 50 349–357. 10.1002/ana.1096 11558791

[B78] Waxman S. G. (2006). Ions, energy and axonal injury: towards a molecular neurology of multiple sclerosis. *Trends Mol. Med.* 12 192–195. 10.1016/j.molmed.2006.03.001 16574486

[B79] Whitney L. W. Becker K. G. Tresser N. J. Caballero-Ramos C. I. Munson P. J. Prabhu V. V. (1999). Analysis of gene expression in mutiple sclerosis lesions using cDNA microarrays. *Ann. Neurol.* 46 425–428. 10.1002/1531-8249(199909)46:3<425::AID-ANA22>3.0.CO;2-O 10482277

[B80] Wiedemann F. R. Winkler K. Kuznetsov A. V. Bartels C. Vielhaber S. Feistner H. (1998). Impairment of mitochondrial function in skeletal muscle of patients with amyotrophic lateral sclerosis. *J. Neurol. Sci.* 156 65–72. 10.1016/S0022-510X(98)00008-2 9559989

[B81] Wingerchuk D. M. Lennon V. A. Pittock S. J. Lucchinetti C. F. Weinshenker B. G. (2006). Revised diagnostic criteria for neuromyelitis optica. *Neurology* 66 1485–1489. 10.1212/01.wnl.0000216139.44259.74 16717206

[B82] Wong P. C. Pardo C. A. Borchelt D. R. Lee M. K. Copeland N. G. Jenkins N. A. (1995). An adverse property of a familial ALS-linked SOD1 mutation causes motor neuron disease characterized by vacuolar degeneration of mitochondria. *Neuron* 14 1105–1116. 10.1016/0896-6273(95)90259-7 7605627

[B83] Xiao B. G. Zhang G. X. Ma C. G. Link H. (1996). The cerebrospinal fluid from patients with multiple sclerosis promotes neuronal and oligodendrocyte damage by delayed production of nitric oxide in vitro. *J. Neurol. Sci.* 142 114–120. 10.1016/0022-510X(96)00164-5 8902730

[B84] Yurube T. Takada T. Hirata H. Kakutani K. Maeno K. Zhang Z. (2011). Modified house-keeping gene expression in a rat tail compression loading-induced disc degeneration model. *J. Orthop. Res.* 29 1284–1290. 10.1002/jor.21406 21387398

[B85] Zhong H. Simons J. W. (1999). Direct comparison of GAPDH, beta-actin, cyclophilin, and 28S rRNA as internal standards for quantifying RNA levels under hypoxia. *Biochem. Biophys. Res. Commun.* 259 523–526. 10.1006/bbrc.1999.0815 10364451

[B86] Zhou L. Lim Q. E. Wan G. Too H. P. (2010). Normalization with genes encoding ribosomal proteins but not GAPDH provides an accurate quantification of gene expressions in neuronal differentiation of PC12 cells. *BMC Genomics* 11:75 10.1186/1471-2164-11-75 PMC2831847 20113474

[B87] Zhu X. Perry G. Moreira P. I. Aliev G. C. Cash A. D. Hirai K. (2006). Mitochondrial abnormalities and oxidative imbalance in Alzheimer disease. *J. Alzheimers Dis.* 9 147–153. 10.3233/JAD-2006-9207 16873962

